# Negative Academic Emotion and Psychological Well-being in Chinese Rural-to-Urban Migrant Adolescents: Examining the Moderating Role of Cognitive Reappraisal

**DOI:** 10.3389/fpsyg.2017.01312

**Published:** 2017-08-02

**Authors:** Daoyang Wang, Shuting Li, Mingming Hu, Dan Dong, Sha Tao

**Affiliations:** ^1^Collaborative Innovation Center of Assessment toward Basic Education Quality, Beijing Normal University Beijing, China; ^2^Department of Psychology, Anhui Normal University Wuhu, China; ^3^State Key Laboratory of Cognitive Neuroscience and Learning, Beijing Normal University Beijing, China; ^4^IDG/McGovern Institute for Brain Research, Beijing Normal University Beijing, China

**Keywords:** rural-to-urban migrant adolescents, negative academic emotions, psychological well-being, cognitive reappraisal, emotion regulation strategies

## Abstract

The study aimed to explore the relationship among negative academic emotions (e.g., anxiety, shame, anger, boredom, hopelessness, disappointment, and hatred), psychological well-being (including life vitality, health concern, altruism commitment, self-value, friendly relationship, and personal development), and cognitive reappraisal in rural-to-urban migrant adolescents in China. Specifically, it was hypothesized that the relationship between psychological well-being and negative academic emotions is moderated by cognitive reappraisal. A total of 311 migrant adolescents aged 14–20 years were selected, including 132 boys and 179 girls. Results of a regression analysis showed that cognitive reappraisal (positive) and negative academic emotions were significant predictors of psychological well-being. The interaction effect between cognitive reappraisal and negative academic emotion was also a significant predictor of psychological well-being. In the simple slope analysis the group with a below average cognitive reappraisal score the negative academic emotions were associated with lower psychological well-being, whereas in the group with above average cognitive reappraisal the effect of negative academic emotions on psychological well-being was not significant. However, for those with a cognitive reappraisal score of 1 standard deviation above the average, the effect of negative academic emotions on psychological well-being was not significant. These results suggest that cognitive reappraisal was a significant moderator in the relationship between negative academic emotion and psychological well-being.

## Introduction

During the past three decades, China has probably experienced the largest peacetime population movement in history (Jin et al., 2012). China’s migrant population has reached 221 million, accounting for 17% of the total population in 2011 (Hu H. et al., 2014; Hu S. et al., 2014). In 2013, approximately 35.8 million migrant children and adolescents resided in China (Hu H. et al., 2014). Rural-to-urban migrant children and adolescents are often students aged 7–18 years, whose parents or other guardians maintain a household in rural areas, but who live for 3 months or more in a different city (Ye et al., 2016). The rural-to-urban migrants definition is important, as it provides a distinction between urban migrants moving to either another urban area or a rural area outside of their current residence.

Rural-to-urban migrant children may change their lifestyle and habits with impacts of the urban life. There are striking social and cultural differences between urban and rural areas in China. Compared to urban students, rural-to-urban migrant students may have more emotional disorders (such as tiredness, disappointment, and anxiety) when trying to adapt to the urban life (Chen, 2014). Parental academic involvement (academic socialization, home-based involvement, and school-based involvement) may also contribute to the inferior status of school functions among rural-to-urban migrant children (Fang et al., 2017). In addition, the lower family socioeconomic status (SES) (e.g., family income and father’s/mother’s educational level) of rural-to-urban children may contribute to such differences (Huang et al., 2017). The rural-to-urban migrant student’s family SES may be improved significantly by moving from rural to urban areas. And increasing family SES may effectively improve the rural-to-urban migrants’ well-being (Huang et al., 2017). The strengthen of family SES will bring the improvement of rural-to-urban migrant students’ studying and living conditions and promote them to adapt to urban life quickly. However, it shall be recognized that the rural-to-urban migrant students may still stand on the bottom of the social ladder when being compared with their urban peers (Huang et al., 2017). Therefore, rural-to-urban migrant students may still have more emotional disorders.

In fact, the migration may result in poor learning and may produce different levels of emotional disturbance in adolescents (Kouider et al., 2015). Research has examined adolescents’ academic life and emotions, which indicated that they experience various emotions in the process of teaching and learning, including happiness, tiredness, disappointment, anxiety, and anger (Pekrun et al., 2002). Dong and Yu (2007) reported that adolescents’ negative academic emotions included high arousal negative academic emotion and low arousal negative academic emotion. High arousal negative academic emotions include anxiety, shame, and anger, while low arousal academic emotions include boredom, hopelessness, disappointment, and hatred. However, migrant adolescents’ academic performance is promoted by mainly focusing on reducing negative academic emotion (Ainley et al., 2005). Lin et al. (2009) suggested that, compared with non-migrant urban peers, migrant children suffer from a higher prevalence of emotional problems, including depression and social anxiety.

Psychological well-being and more positive or less negative emotions are closely related. Psychological well-being is seen as the absence of illness or the presence of positive subjective feelings about oneself (Ryff and Singer, 2008). In Chinese cultural contexts, Miao (2003, Unpublished) reported that psychological well-being has six dimensions, including life vitality, health concern, altruism commitment, self-value, friendly relationship, and personal development. One study suggested that the presence of psychological distress is associated with negative emotions and diminishes psychological well-being during adolescence (Cripps and Zyromski, 2009). Further, Urry et al. (2004) suggested that psychological well-being is related to positive affect. The limitation of existing studies on this topic is that most have been conducted in Western cultures (To et al., 2017). Further, the few studies conducted in the Chinese cultural context have focused almost exclusively on urban or rural adolescents. However, it is also important to focus on the rural-to-urban migrant student’s psychological well-being.

Appraisal theories of emotion suggest that appraisal is a component of an emotional episode, and that appraisal theories can account for differences in people’s emotional responses to the same situation (Moors et al., 2013). Because appraisals appear to play an important role in the generation of emotional states, emotion regulation strategies that target appraisals should be particularly effective (Troy et al., 2010). Cognitive reappraisal refers to reframing an event in order to change one’s emotional response to it Gross (1998). Appraisal is a process that detects and assesses the significance of the environment for well-being (Frijda, 2009). Further, Gross and John (2003) suggested that emotion regulation is involved in well-being. Because individuals often use cognitive reappraisal to regulate their academic emotions, cognitive reappraisal may be associated with lower level of negative academic emotions and may contribute to migrant student’s psychological well-being in the Chinese context.

Therefore, the present study examined the relationship among psychological well-being, negative academic emotions, and cognitive reappraisal in rural-to-urban migrant adolescents. The following hypotheses were formulated in order to achieve the goals of this research: (a) cognitive reappraisal and negative academic emotions are significant predictors of psychological well-being, and (b) cognitive reappraisal moderates the relationship between negative academic emotions and psychological well-being.

## Materials and Methods

### Participants and Procedure

The survey was conducted between March and April 2016. Participants were 877 adolescents enrolled at two middle schools and two colleges in Anhui and Beijing, China. Each class was selected randomly at each grade level. The final selection of 22 classes, including middle school 12 classes, 10 classes in the college. For this study, data was obtained via the Internet because online versions of personality questionnaires have good equivalence and similar psychometric properties to traditional paper and pencil forms (Gosling et al., 2004). Written consent was obtained from each participant after a full explanation of the study procedure was provided. Parents/guardians of participants aged below 18 years were informed, and their consent was obtained.

The following eligibility criteria were used to select rural-to-urban migrant adolescents: (a) no household registered (i.e., *hukou*) in urban areas (e.g., Beijing, Hefei, and Wuhu) and (b) temporarily living with parents who have migrated to an urban area and have lived there for more than 3 months. Finally, the sample consisted of 311 Chinese adolescents aged 14–20 years. For those, 42.4% were boys (*n* = 132) and 57.6% were girls (*n* = 179). The mean age for males was 18.69 ± 1.04 years. Their fathers’ years of education ranged from 0 to 18 (*M* = 8.42, *SD* = 3.08), and mothers’ years of education ranged from 0 to 18 (*M* = 6.65, *SD* = 3.32). Their annual family income ranged from 1 to 9 (see Background Questionnaire), the *M* and *SD* were 3.57 and 1.74, respectively.

### Instruments

#### Background Questionnaire

The Background Questionnaire is a self-reported inventory, and it includes gender, date of birth, annual family income [In last year, your family income (RMB) per capital was about......? 1 = ¥ 3000, 2 = ¥ 3001 ∼ 6000, 3 = ¥ 6001 ∼ 10000, 4 = ¥ 10001 ∼ 30000, 5 = ¥ 30001 ∼ 50000, 6 = ¥ 50001 ∼ 100000, 7 = ¥ 100001 ∼ 150000, 8 = 150001 ∼ 200000, 9 = more than ¥ 200001], and father’s/mother’s educational level (i.e., years of education).

#### Psychological Well-being Questionnaire

The Psychological Well-being Questionnaire has been developed by Miao (2003, Unpublished). This 33 item questionnaire measures psychological well-being through six subscales, including life vitality (six items, e.g., “I am young and full of energy”), health concern (five items, e.g., “I have a healthy lifestyle”), altruism commitment (five items, e.g., “I help others when they are in need”), self-value (five items, e.g., “I’m satisfied with my situation for now”), friendly relationship (three items, e.g., “I have a close friends”), and personal development (nine items, e.g., “I possess an ability to understand and accept myself”). Respondents answer each item on a seven-point Likert-type scale ranging from 1 (strongly disagree) to 7 (strongly agree). The respective Cronbach’s coefficient and split-half reliability scores for each subscale were as follows: life vitality (0.84 and 0.80), health concern (0.76 and 0.74), altruism commitment (0.84 and 0.83), self-value (0.76 and 0.74), friendly relationship (0.91 and 0.88), and personal development (0.67 and 0.62).

#### Adolescent Negative Academic Emotion Questionnaire

The Adolescent Academic Emotion Questionnaire (Dong and Yu, 2007) included a total of four subscales: high arousal positive academic emotions, low arousal positive academic emotions, high arousal negative academic emotions, and low arousal negative academic emotions. Our study used all the items of the negative academic emotion subscale (high arousal and low arousal), and excluded all items of the positive academic emotion subscale. High arousal negative academic emotions include anxiety, shame, and anger (total 17 items, e.g., “I feel irritated while studying”), and low arousal academic emotions include boredom, hopelessness, disappointment, and hatred (total 25 items, e.g., “I find it boring to study math or English”). Each item was a statement that described an emotion, and the participants were asked to indicate the extent to which each statement generally described them, using a five-point Likert-type scale ranging from 1 (*totally not in line with*) to 5 (*fully in line with*). The Cronbach’s coefficients for the two subscales (high arousal and low arousal) of negative academic emotions were 0.83 and 0.92, and the split-half reliabilities were 0.79 and 0.82, respectively.

#### Cognitive Reappraisal Scale

Cognitive reappraisal was measured using the six item, reappraisal subscale of the Emotion Regulation Questionnaire (Gross and John, 2003), which comprises items such as, “When I want to feel more *positive* emotions (such as joy or amusement), I *change what I’m thinking* about,” “ When I want to feel less *negative* emotion (such as sadness or anger), I *change what I’m thinking* about,” “When I’m faced with a stressful situation, I make myself *think about it* in a way that helps me stay calm,” “When I want to feel more *positive* emotions, I *change the way* I’m thinking about the situation,” “I control my emotions by *changing the way* I think about the situation I’m in,” and “When I want to feel less *negative* emotions, I *change the way* I’m thinking about the situation.” Respondents answer each item on a seven-point Likert-type scale ranging from 1 (strongly disagree) to 7 (strongly agree), and the internal consistency alpha of the cognitive reappraisal subscale was 0.82.

### Statistical Analyses

All statistical analyses were conducted using SPSS 22.0 (SPSS, Chicago, IL, United States). First, the relationships among psychological well-being, cognitive reappraisal, negative academic emotions, gender, age, annual family income, and father’s/mother’s educational level were established using Pearson’s correlations. Subsequently, a regression analysis was performed to test if cognitive reappraisal was a moderator in the relationship between negative academic emotions and psychological well-being. In the analysis, cognitive reappraisal (± 1 *SD*), negative academic emotions, and their interaction term (cognitive reappraisal × negative academic emotions) were the independent variables, and psychological well-being was the dependent variable. Gender, age, annual family income, and father’s/mother’s educational level were used as control variables. Moreover, simple slope analyses were used to explore the interaction effect.

## Results

### Descriptive Statistics

The descriptive statistics for gender, age, the SES variables (i.e., annual family income and father’s and mother’s educational level), psychological well-being, cognitive reappraisal, and negative academic emotions, and their correlations have been described in **Table 1**. Psychological well-being was positively correlated with cognitive reappraisal (*r* = 0.367, *p* < 0.01) and this correlation remained significant when controlling for gender, age, and SES (*r* = 0.326, *p* < 0.01). Negative academic emotions was negatively significantly correlated with psychological well-being (*r* = 0.112, *p* < 0.05) and the correlations remained non-significant when controlling for gender, age, and SES (*r* = 0.106, *p* = 0.081). Cognitive reappraisal was non-significantly correlated with negative academic emotions (*r* = 0.188, *p* < 0.01) and the correlations remained significant when controlling for gender, age, and SES (*r* = 0.141, *p* < 0.05). Furthermore, gender, age, annual family income, and father’s/mother’s educational level had either weak or non-significant correlations with psychological well-being, cognitive reappraisal, and negative academic emotions.

**Table 1:** Descriptive statistics and Pearson correlations.

	*M*	*SD*	1	2	3	4	5	6	7
(1) Gender									
(2) Age	18.69	1.04	-0.063	—					
(3) Annual family income	3.57	1.74	-0.122^∗^	-0.155^∗∗^	—				
(4) Father’s educational level	8.42	3.08	0.306^∗∗^	0.212^∗∗^	0.045	—			
(5) Mother’s educational level	6.65	3.32	0.080	0.081	0.152^∗∗^	0.485^∗∗^	—		
(6) Psychological well-being	117.21	32.17	0.044	0.051	0.030	-0.037	-0.098	—	
(7) Cognitive reappraisal	20.13	6.36	0.006	0.027	0.029	-0.069	-0.114^∗∗^	0.367^∗∗^	—
(8) Negative academic emotion	127.40	23.33	-0.071	0.044	-0.024	0.070	0.091	-0.112^∗^	0.188^∗∗^

*^∗^*p* < 0.05; ^∗∗^*p* < 0.01.*

### Moderator Analyses

Regression analyses were performed to test the moderating effect of cognitive reappraisal on the relationship between negative academic emotions and psychological well-being. In the first step, cognitive reappraisal, negative academic emotions, and the five control variables (i.e., gender, age, and SES variables) were added as independent variables. In the second step, the interaction between centerized cognitive reappraisal and negative academic emotion was included. The results of the regression analyses have been depicted in **Table 2**. In Model 1, cognitive reappraisal (β = 0.331, *p* < 0.001) and negative academic emotion (β = -0.149, *p* < 0.01) were revealed as significant predictor of psychological well-being. The controlled variables (i.e., gender, annual family income, and father’s/mother’s educational level) had no significant influence on psychological well-being, except age (β = 0.126, *p* < 0.05).

**Table 2:** Summary of regression analysis for variables predicting psychological well-being without interaction term (Model 1) and with interaction term (Model 2).

Variable	Model 1			Model 2		
	*B*	SE B	β	*B*	SE B	β
Gender	2.113	3.168	0.038	2.984	3.149	0.054
Age	1.785	0.793	0.126^∗^	1.319	0.802	0.093
Annual family income	0.469	0.785	0.033	0.279	0.779	0.020
Father’s educational level	-0.242	0.533	-0.030	-0.396	0.530	-0.049
Mother’s educational level	-0.108	0.507	-0.014	-0.048	0.502	-0.006
Cognitive reappraisal	1.606	0.265	0.331^∗∗∗^	1.270	0.290	0.262^∗∗^
Negative academic emotion	-0.179	0.066	-0.149^∗∗^	-0.148	0.067	-0.124^∗^
Cognitive Reappraisal × Negative academic emotion				-0.024	0.009	-0.170^∗∗^
*R*^2^		0.174			0.195	
*F*		8.564^∗∗∗^			8.590^∗∗∗^	

*^∗^*p* < 0.05; ^∗∗^*p* < 0.01; ^∗∗∗^*p* < 0.001.*

In Model 2, cognitive reappraisal (β = 0.262, *p* < 0.001) and negative academic emotions (β = -0.124, *p* < 0.05) were revealed as significant predictors of psychological well-being. Moreover, the interaction effect between cognitive reappraisal and negative academic emotions (β = 0.170, *p* < 0.01) was a significant predictor of psychological well-being. The *R*^2^-change (ΔR^2^) between Model 1 and Model 2 was significant (ΔR^2^ = 0.021, *p* < 0.01). The interaction term in Model 2 was significant (β = 0.170, *t* = 2.723, *p* < 0.01), which indicated that cognitive reappraisal was a significant moderator in the relationship between negative academic emotions and psychological well-being.

**FIGURE 1:**
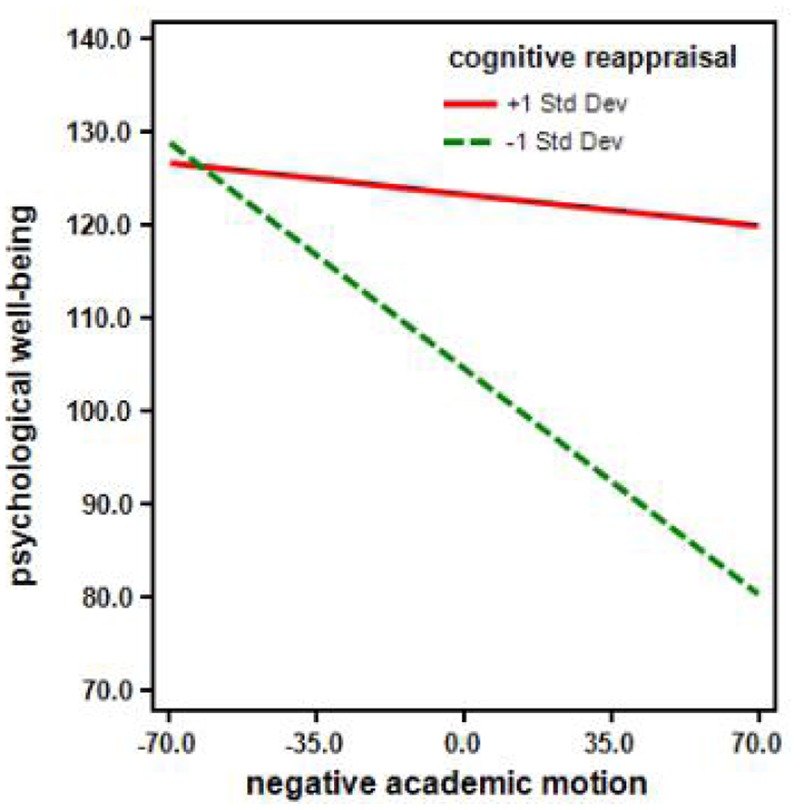
Plots of slopes for the interaction between negative academic emotion and cognitive reappraisal (±1 SD) on psychological well-being, using gender, age, and SES as control variables.

To further investigate the nature of this moderation, a simple slope analysis was performed separately for cognitive reappraisal at ±1 *SD* from the mean (**Figure 1**). In this figure, the downward slope represents the relationship between negative academic emotions and psychological well-being when cognitive reappraisal was below average-specifically, when the z value of cognitive reappraisal was 1 *SD* below the average. The other almost horizontal line represents the relationship between negative academic emotions and psychological well-being when cognitive reappraisal was above average-specifically, when the z value of cognitive reappraisal was 1 *SD* above the average.

These analyses revealed that, in those with a cognitive reappraisal score of 1 *SD* below the average, there was a significant relationship between negative academic emotions and psychological well-being (simple slope = -0.348, *t* = -7.83, *p* < 0.001), indicating that higher negative academic emotions were associated with lower psychological well-being. However, for those with a cognitive reappraisal score of 1 *SD* above the average, the effect of negative academic emotions on psychological well-being was not significant (simple slope = -0.049, *t* = -1.06, *p* = 0.144).

## Discussion

In the present study on rural-to-urban migrant adolescents, negative academic emotions, psychological well-being, and cognitive reappraisal were assessed. The results revealed that cognitive reappraisal moderates the relationship between negative academic emotions and psychological well-being in rural-to-urban migrant adolescents.

Specifically, in the present study, psychological well-being was positively correlated with cognitive reappraisal, and negative academic emotions were not significantly correlated with psychological well-being. The link between cognitive reappraisal and psychological well-being has already been suggested by previous research. For example, Balzarotti et al. (2016) showed that positive reappraisal and refocus on planning are positively related to psychological well-being. Meanwhile, the present regression analyses suggested that cognitive reappraisal can predict psychological well-being. Again, this finding had been supported by previous studies. For instance, research has shown that people who engage in cognitive reappraisal more frequently enjoy positive mental health related outcomes (Ranney et al., 2016), greater psychological well-being (Shiota, 2006), less negative emotions, and more positive emotions (Gross and John, 2003).

In addition, cognitive reappraisal was a significant moderator of the relationship between negative academic emotions and psychological well-being. These analyses revealed that, in those with a cognitive reappraisal score of 1 *SD* below the average, higher negative academic emotions were associated with lower psychological well-being. However, in those with a cognitive reappraisal score of 1 *SD* above the average, the effect of negative academic emotions on psychological well-being was not significant. These results indicated that higher cognitive reappraisal can weaken the negative effect of negative academic emotions on psychological well-being. Emotional regulation through cognitive reappraisal pertains to the conscious thoughts by means of which individuals regulate their emotions in response to adverse events (Garnefski et al., 2002). This has a very real significance for the regulation negative academic emotions for rural-to-urban migrant adolescents. For instance, they may reframe the meaning of a harmful event while adapting to school life.

In summary, the present study found cognitive reappraisal significantly moderated the association between negative academic emotions and psychological well-being in rural-to-urban migrant adolescents. The rural-to-urban migrant students’ problems in their adaption may be not only linked to their emotional distress, but also to lower academic achievement and poorer psychological well-being, higher negative emotions and more truancy. The educational success of rural-to-urban migrant students is not only critical for individual upward mobility, but also has a fundamental impact on society’s economic well-being and social stability (Fang et al., 2017). The findings from the present study may suggest some strategies to help the rural-to-urban migrant students. On the one hand, the school may provide rural-to-urban migrant students with the training on emotional regulation. For example, cognitive reappraisal strategies may be instructed and trained among migrant students. On the other hand, the local governments and schools may help the rural-to-urban migrant students adapt to urban life as soon as possible by facilitating exchanges and mutual understanding with urban students. For instance, the state may have public schools accessible to rural-to-urban migrant students, and may provide schools with necessary financial support and other resources to promote the well-being of migrant children (Li et al., 2010).

A number of limitations of the present study need to be mentioned. First, the purpose of this study was to investigate the rural-to-urban migrant adolescents, but not to compare them with typical urban adolescents and rural adolescents, and the role of school factors, academic performance, and other factors were not considered. Thus, the results are limited in scope, and further in-depth study is necessary. Secondly, the sample consisted of migrant adolescents only from Anhui and Beijing. Future studies could also be improved by recruiting more migrant adolescents from other areas in China. Finally, there was lack of experimental control of migration time variables, and self-report measures were used for assessing cognitive reappraisal and negative academic emotions.

Further research may take the following aspects into consideration. First, not only self-report questionnaires but also physiological measures, biochemical examination, and direct behavioral test of emotion shall be used to reveal a full picture of negative academic emotion on psychological well-being (Ong et al., 2006). Second, the comparisons between rural and urban children shall go in deep to identify if cognitive reappraisal for negative academic emotions may develop and impact in different ways, and to find out the effective strategies stratified for various groups. Third, whether and how the moderation effect occurs in various dimensions of psychological well-being merit further examination.

## Ethics Statement

The study was reviewed and approved by the Institutional Review Board of Human Research Ethics Committee for Non-Clinical Faculties of Collaborative Innovation Center of Assessment toward Basic Education Quality (CICA-BEQ) at Beijing Normal University.

## Author Contributions

DW drafted the work, which was revised critically by ST, SL, and MH. ST contributed to all steps of the work. SL and DD contributed to the interpretation of the data for the work. All authors approve of the final version of the manuscript and agree to be accountable for all aspects of the work in ensuring that questions related to the accuracy or integrity of any part of the work are appropriately investigated and resolved.

## Conflict of Interest Statement

The authors declare that the research was conducted in the absence of any commercial or financial relationships that could be construed as a potential conflict of interest. The reviewer MD and handling Editor declared their shared affiliation, and the handling Editor states that the process nevertheless met the standards of a fair and objective review.
